# Supplementary material for: Reconciling Mining with the Conservation of Cave Biodiversity: A Quantitative Baseline to Help Establish Conservation Priorities
Source: PLoS One. 2016 Dec 20;11(12):e0168348. doi: 10.1371/journal.pone.0168348 (PMC5173368; doi:10.1371/journal.pone.0168348)
Supplement: S1 Dataset — (ZIP) [file pone.0168348.s002.zip › Taxa/Serra Norte/SN_2013/N4E/Lista N4E-12.pdf]

CAVIDADE N4E-0012

Classe	Ordem	Fam/Outros	Gên/Outros	Espécie	S-U	U-S	Zon.
Arachnida	Acari	Trombiculidae		sp.	X		
Arachnida	Amblypygi	Charinidae	*Charinus*	sp.1		1	P
Arachnida	Amblypygi	Phrynidae	*Heterophrynus*	*longicornis*	X		
Arachnida	Araneae	Corinnidae		jovem		1	P
Arachnida	Araneae	Ctenidae		jovem	1		
Arachnida	Araneae	Drymusidae		jovem		X	P
Arachnida	Araneae	Filistatidae		jovem	X	X	P
Arachnida	Araneae	Ochyroceratidae	*Ochyrocera*	sp.	X	X	
Arachnida	Araneae	Pholcidae	*Mesabolivar*	sp.1	X	X	P
Arachnida	Araneae	Pholcidae	Ninetinae	sp.1		X	P
Arachnida	Araneae	Scytodidae	*Scytodes*	*eleonorae*		1	P
Arachnida	Araneae	Theridiidae	*Theridion*	sp.1		X	E
Arachnida	Araneae	Theridiidae	*Thymoites*	sp.	X		
Arachnida	Araneae	Theridiosomatidae	*Ogulnius*	sp.1		X	E
Arachnida	Araneae	Theridiosomatidae	*Plato*	sp.1		X	P
Arachnida	Opiliones	Stygnidae	*Protimesius*	*laevis*	X		
Arachnida	Pseudoscorpiones	Chthoniidae		sp.	X		
Arachnida	Pseudoscorpiones	Olpiidae		jovem	X	X	P
Chilopoda	Geophilomorpha			jovem	X		
Chilopoda	Scolopendromorpha	Cryptopidae	*Cryptops*	jovem		1	P
Entognatha	Collembola	Paronellidae		sp.1	X	X	P
Entognatha	Diplura	Campodeidae		sp.1	X		
Entognatha	Diplura	Projapygidae		sp.1		X	P
Insecta	Blattodea	Polyphagidae		jovem	1	3	E
Insecta	Coleoptera	Chelonariidae		sp.1	X		
Insecta	Coleoptera	Lampyridae		jovem	X		
Insecta	Coleoptera	Staphylinidae	Pselaphinae	sp.8		X	P
Insecta	Coleoptera	Staphylinidae		sp.1	X	X	E
Insecta	Diptera	Psychodidae	Phlebotominae	sp.		X	E
Insecta	Diptera			sp.	X	X	E/P
Insecta	Heteroptera	Cydnidae		sp.	X		
Insecta	Heteroptera	Reduviidae	Emesinae	sp.		2	P
Insecta	Heteroptera	Reduviidae	*Ploiaria*	sp.	X		
Insecta	Homoptera	Cixiidae		jovem	X		
Insecta	Hymenoptera	Formicidae	*Camponotus*	*atriceps*	X		
Insecta	Hymenoptera	Formicidae	*Gnamptogenys*	*striatula*	X		
Insecta	Hymenoptera	Formicidae	*Pachycondyla*	sp.2		X	E
Insecta	Hymenoptera	Formicidae	*Pheidole*	sp.2		X	P
Insecta	Hymenoptera	Formicidae	*Pheidole*	sp.3		X	P
Insecta	Hymenoptera	Formicidae	*Solenopsis*	sp.	X		
Insecta	Hymenoptera	Sphecidae		sp.	X		
Insecta	Isoptera			sp.		X	P
Insecta	Lepidoptera	Noctuoidea		sp.1		1	P
Insecta	Lepidoptera	Tineoidea		sp.1	X	X	P
Insecta	Neuroptera	Myrmeleontidae		jovem		X	P
Insecta	Orthoptera	Phalangopsidae	*Paraclodes*	jovem		1	P
Insecta	Orthoptera	Phalangopsidae	*Phalangopsis*	sp.	X		
Insecta	Psocoptera	Manicapsocidae	*Nothoentomum*	sp.1		X	E
Insecta	Psocoptera	Psocomorpha		jovem	X		
Insecta	Psocoptera	Psyllipsocidae		jovem		X	P
Malacostraca	Isopoda	Armadillidae		sp.1			
Malacostraca	Isopoda	Philosciidae		sp.	X		
Symphyla		Scutigerellidae		sp.		X	P
Amphibia	Anura	Leptodactylidae		sp.	1		P
Amphibia	Anura			sp.		4	E/P

Mammalia	Chiroptera	Emballonuridae	*Peropteryx*	sp.		5	E/P
